# Supplementary material for: Novel Fluorescent Strategy for Discriminating T and B Lymphocytes Using Transport System
Source: Pharmaceutics. 2024 Mar 19;16(3):424. doi: 10.3390/pharmaceutics16030424 (PMC10974409; doi:10.3390/pharmaceutics16030424)
Supplement: Supplementary file 1 [file pharmaceutics-16-00424-s001.zip › pharmaceutics-2904484-supplementary.pdf]

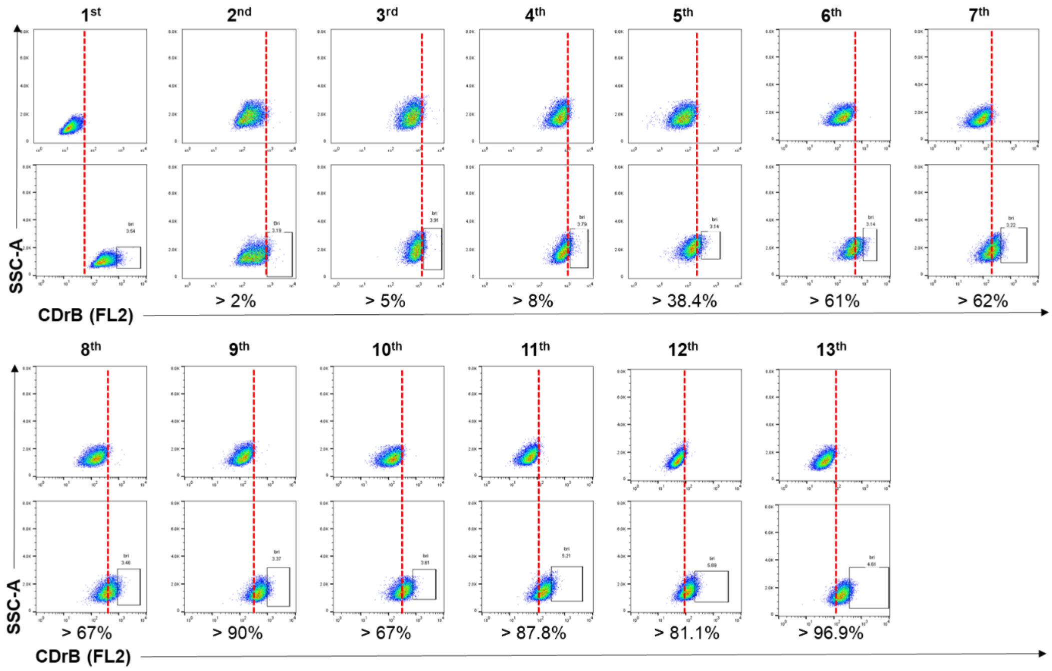

Figure S1. Fluorescence enrichment of the CDrB stained SLC-CRISPRa and sorted out brighter populations. SLC-CRISPRa library was stained by **CDrB** (0.5 µM). After 30 min, ~3% brigher populations were sorted out, and repeated processes until ~95% enrichment compared to control library. The gating strategy was described in Materials and methods section.

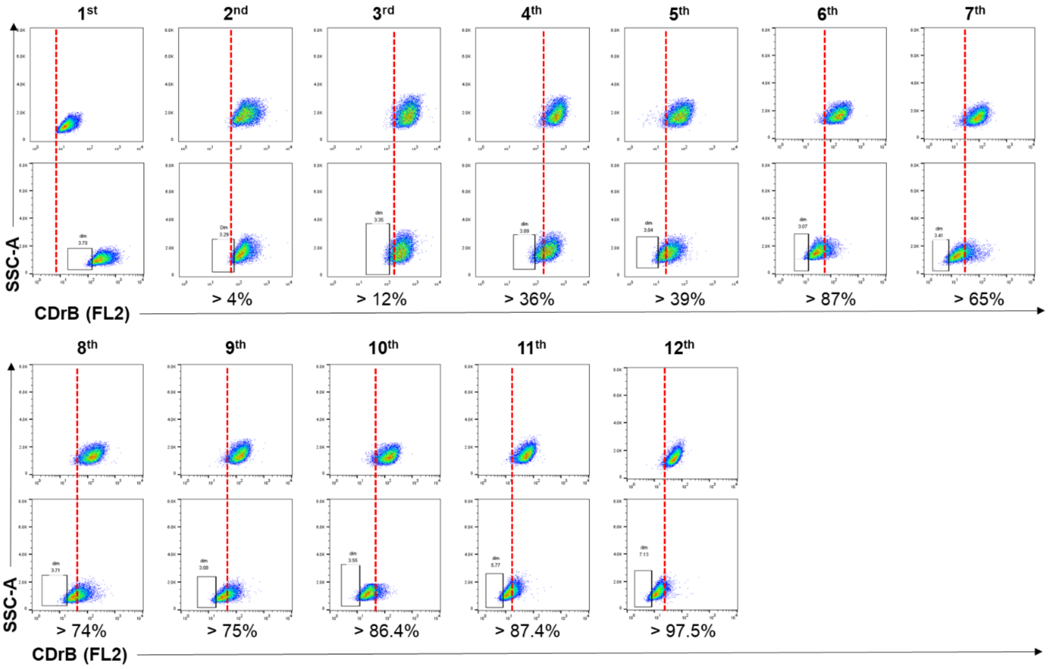

Figure S2. Fluorescence enrichment of the CDrB stained SLC-CRISPRa and sorted out dimmer populations. SLC-CRISPRa library was stained by **CDrB** (0.5 µM). After 30 min, ~3% dimmer populations were sorted out, and repeated processes until ~95% enrichment compared to control library. The gating strategy was described in Materials and methods section.

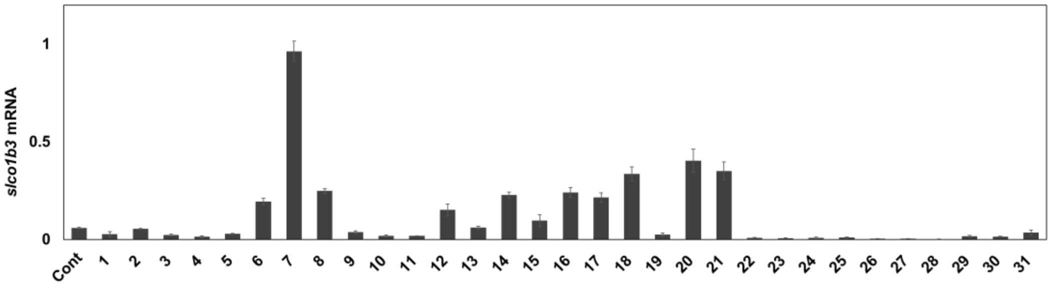

Figure S3. Single cell cloning for slco1b3. After enrichment of brigher populations in SLC-CRISPRa pools, single cell cloning was carried out. 31 clones were identified, and 7 clone showed a higher expression level of *slco1b3* compared to others.

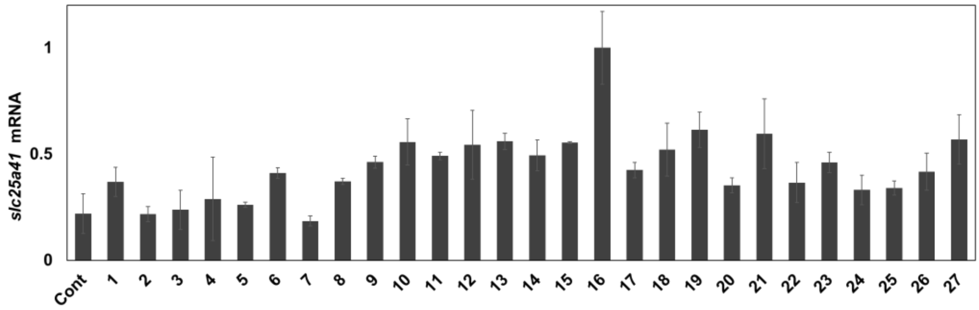

Figure S4. Single cell cloning for slc25a41. After enrichment of dimmer populations in SLC-CRISPRa pools, single cell cloning was carried out. 31 clones were identified, and 16 clone showed a higher expression level of *slc25a41* compared to others.

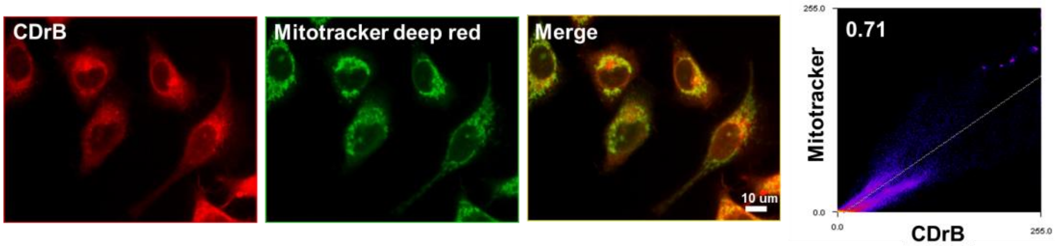

Figure S5. Localization of CDrB. The cells were stained with Mitotracker deep red (100 nM) after staining **CDrB** (1 μM). The correlation graph was displayed with Pearson's correlation coefficient (0.71)

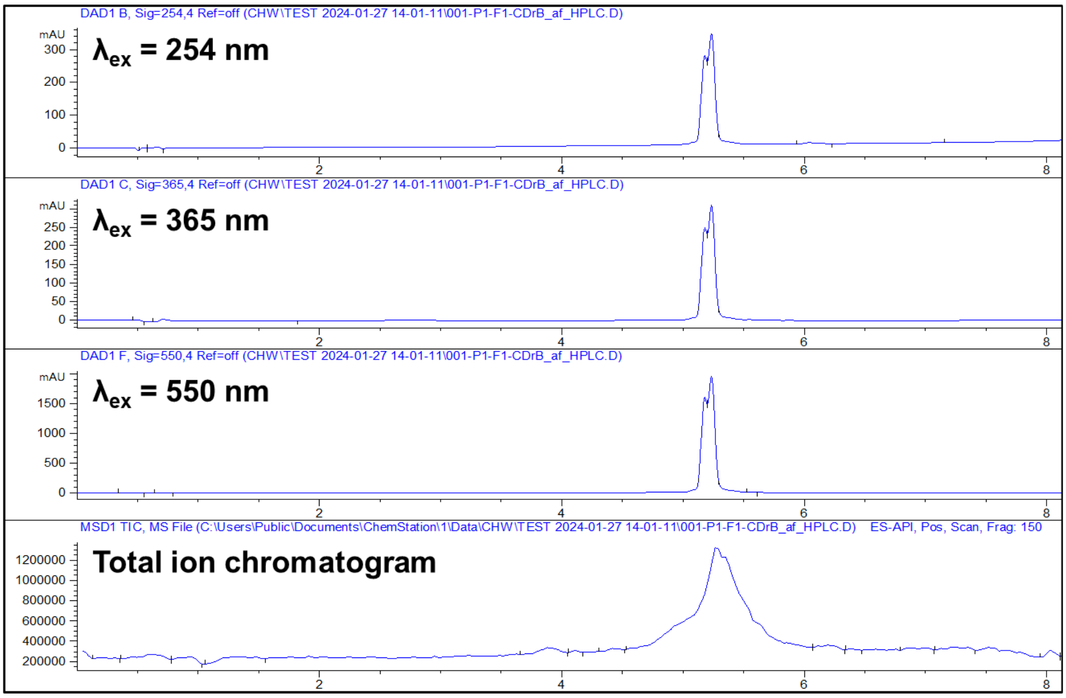

Figure S6. LC and mass chromatogram of CDrB.

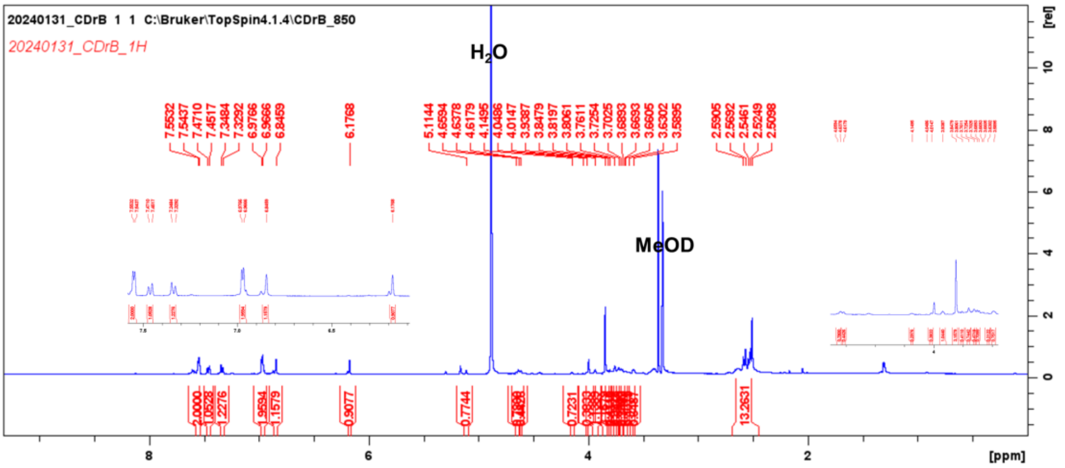

Figure S7. ^{1}H NMR of CDrB (850 MHz, MeOH-*d4*).

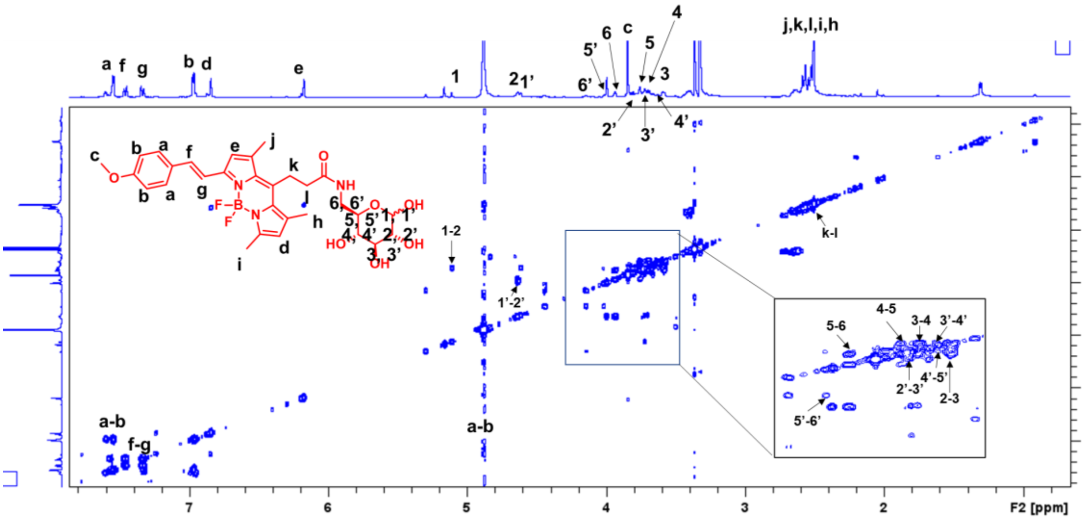

Figure S8. ^{1}H-^{1}H COSY of CDrB (850 MHz, MeOH-*d4*).

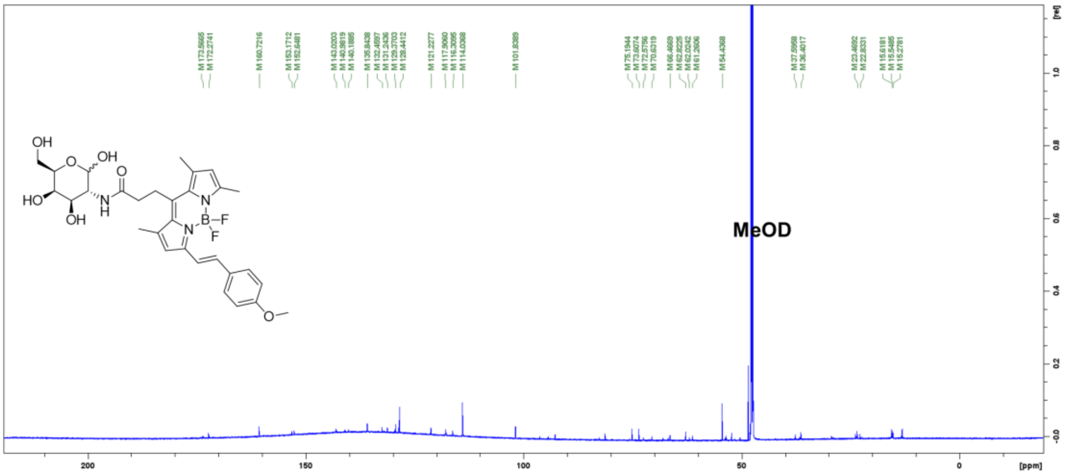

Figure S9. ^{13}C NMR spectrum of CDrB (214 MHz, MeOH-*d4*).

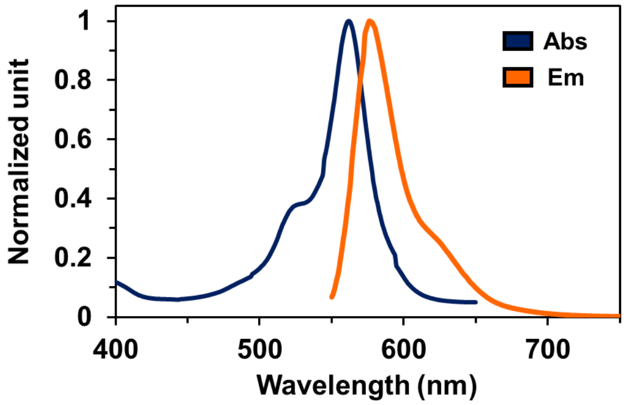

λ_{abs}	λ_{em}	ε ($cm^{-1}M^{-1}$)	Φ_{fl}
562 nm	576 nm	73,080	0.28

Figure S10. Absorbance and fluorescence spectra of CDrB in MeOH. The displayed graph showed the absorbance and emission spectrum of **CDrB**. The table contained the information of extinction coefficient and quantum yield of **CDrB**, which were measured in methanol. The emission spectrum was measured by λ_{ex} = 550 nm.
